# Cortical Thymocytes Along With Their Selecting Ligands Are Required for the Further Thymic Maturation of NKT Cells in Mice

**DOI:** 10.3389/fimmu.2020.00815

**Published:** 2020-05-07

**Authors:** Jihene Klibi, Kamel Benlagha

**Affiliations:** Université de Paris Diderot, Institut de Recherche Saint Louis (IRSL), Inserm U1160, Paris, France

**Keywords:** NKT1, thymus, development, CD1d, iGb3, cathepsin L, plck

## Abstract

Following positive selection, NKT cell precursors enter an “NK-like” program and progress from an NK^–^ to an NK^+^ maturational stage to give rise to NKT1 cells. Maturation takes place in the thymus or after emigration of NK^–^ NKT cells to the periphery. In this study, we followed the fate of injected NKT cells at the NK^–^ stage of their development in the thymus of a series of mice with differential CD1d expression. Our results indicate that CD1d-expressing cortical thymocytes, and not epithelial cells, macrophages, or dendritic cells, are necessary and sufficient to promote the maturation of thymic NKT1 cells. Migration out of the thymus of NK^–^ NKT cells occurred in the absence of CD1d expression, however, CD1d expression is required for maturation in peripheral organs. We also found that the natural ligand Isoglobotriosylceramide (iGb3), and the cysteine protease Cathepsin L, both localizing with CD1d in the endosomal compartment and crucial for NKT cell positive selection, are also required for NK^–^ to NK^+^ NKT cell transition. Overall, our study indicates that the maturational transition of NKT cells require continuous TCR/CD1d interactions and suggest that these interactions occur in the thymic cortex where DP cortical thymocytes are located. We thus concluded that key components necessary for positive selection of NKT cells are also required for subsequent maturation.

## Introduction

Mouse NKT cells are the product of a special developmental program initiated after their encounter with CD1d molecules presenting self-lipid antigens in the thymus. NKT cells are autoreactive and have been shown to be involved in various conditions such as infectious and autoimmune diseases (1).

Mouse NKT cells were first described as expressing a semi-invariant Vα14-Jα18/Vβ8,Vβ7,Vβ2 αβ T cell receptor (Vα14-Jα18/Vβ TCR) and NK-lineage markers, such as receptors belonging to the CD94/NKG2 or LY49 families (2). In a few mouse strains such as C57BL/6 where NKT cells have been initially extensively studied, they also express the NK1.1 receptor. NKT cells express the activation marker CD44 and also have functional features of NK cells, producing IFN-γ upon stimulation. These cells are fully mature and are at an end, or stage 3, developmental stage. CD1d-tetramers have allowed NKT cells not expressing NK1.1, or NK1.1^–^ NKT cells, to be detected. These cells can express low or high levels of CD44 and represent NKT cells at developmental CD44^low^ stage 1 and CD44^high^ stage 2 (3). Experiments involving thymic transfer of NK1.1^–^ NKT cells showed that these cells are the precursors of NK1.1^+^ NKT cells as they acquired the NK1.1 marker and other NK features (4). The transcription factor T-bet and the cytokine IL-15 were shown to be essential for maturational transition of NKT cells from the NK1.1^–^ to the NK1.1^+^ NKT cell developmental stage (5). This linear model for NKT cell development was established before the discovery of distinct NKT cell subsets classified based on their expression of transcription factors and the cytokines they produce (6). In this classification, the previously described NK1.1^+^ NKT cells expressing T-bet and producing IFN-γ correspond to the NKT1 subset; NKT2 cells express high levels of PLZF and actively produce IL-4; NKT17 cells express RORγt and produce IL-17 and other Th17-related cytokines. Based on these findings, a differentiation model for NKT cell development was proposed in which NKTp represent a precursor cell population of all the NKT cell subsets mentioned above (7).

The development of mouse NKT cells differs in many ways from the development of mainstream T cells. In fact, early studies showed that the Ras/Raf/Mek/MAP pathway was dispensable for the development of NKT cells, but required for mainstream T cell development (8). Other pathways involving the SLAM-SAP-FYN-NF-kB pathway were found to be essential to the development of NKT cells (9). The essence of these differences probably originate from the fact that, unlike mainstream T cells, Vα14 NKT cells are selected by an MHC class I-like molecule that presents agonist self-lipids rather than antagonist peptides (10, 11). Thus, TCR and SLAM appear to provide unique signals driving precursors toward the NKT cell lineage through the expression of the transcription factor PLZF (12). Another unusual aspect of NKT cell development is that the CD1d ligand involved in their positive selection is exclusively presented by bone marrow-derived cells, because NK1.1^+^ cells do not develop in lethally irradiated β2M +/+ mice reconstituted with β2M−/− fetal liver cells, whereas they develop normally in the reciprocal chimeras (13). Double positive thymocytes (DP) are the main cell type mediating the positive selection of Vα14 NKT cells. In fact, Vα14 NKT cell development occurred in mixed chimeras reconstituted with bone marrow cells from CD1d−/− cells (as a source of NKT cells) and TCRα−/− bone marrow cells rather than TCRβ−/− cells (as a source of presenting cells), the latter lack DP cells. These studies were confirmed in mice expressing CD1d only up to the DP stage, thanks to control of its expression under the proximal Lck promoter (14). Interestingly, the Godfrey group (15) showed that, in addition to its role in NKT cell selection, CD1d is also important for the maturation of NKT cells. In fact, NK1.1^–^ NKT cells do not undergo maturational transition when cells are transferred into CD1d−/− mice. These results demonstrate the importance of CD1d/TCR interactions for NKT cell maturation following positive selection. The exact nature of the CD1d-expressing cell type involved in this process has not been established. In addition, whether specific antigens are required during positive selection and maturation remains to be seen.

In this study, we show that thymic transition of NKT cells from the NK^–^ to the NK^+^ stage does not require CD1d expression on specialized APC or on epithelial cells, but that cortical thymocytes play a crucial role by presenting the natural ligand to NKT cells. These cells are involved in the positive selection process, but also continuously promote NKT cell maturation.

## Materials and Methods

### Mice

C57BL/6 (B6) mice, cathepsin L−/− (catL) mice, TCR Cα−/− (Cα−/−) mice on a C57BL/6 background were purchased from Jackson. CD1d−/−, Jα18−/− mice, hexb−/−, pEα - CD1d and pLck-CD1d on B6 background are described elsewhere (14, 16–19). All mice were bred and maintained under specific pathogen-free conditions in compliance with institutional guidelines. Experimental studies were performed in accordance with the Institutional Animal Care and Use Guidelines. The study was approved by the local ethics committee “Comité d’Ethique Paris-Nord; C2EA-121,” affiliated to the “Comité National de Réflexion Ethique en Expérimentation Animale” (CNREEA) and to the French ministry for higher education and research.

### Flow Cytometry

FITC-conjugated mAbs directed against CD44, CD45.1; PE-Cy5-conjugated mAbs against CD24 (HSA); Allophycocyanin (APC)-conjugated mAbs recognizing NK1.1, and PE-conjugated mAbs directed against NK1.1, were obtained from BD PharMingen. CD1d-αGalactosylCeramide (CD1d-αGalCer) tetramers were conjugated with Streptavidin-PE and used for staining as described previously (3). Samples were analyzed using a four-color FACS-Sort^TM^ equipped with argon and 635-nm diode lasers (Becton Dickinson) and CELLQuest^TM^ software.

### FACS Sorting of NKT Cells

Pooled thymuses from batches of 25 to 50 C57BL/6 mice (2- to 4-week-old) were depleted of CD8^+^ cells using anti-CD8 microbeads and the AutoMACs system (Miltenyi Biotec, Auburn, CA), stained with CD1d-αGalCer Streptavidin-PE tetramers and anti-NK1.1. NKT cells were sorted into NK1.1^–^ and NK1.1^+^ subsets. Alternatively, to avoid TCR crosslinking as a result of tetramer staining, CD8-negative thymocyte preparations were stained with HSA, CD44, and NK1.1, and HSA^low^CD44^high^NK1.1^–^ cells were sorted.

### Intrathymic Injection

Sorted tetramer-positive NK1.1^–^, and NK1.1^+^ subsets or CD44^high^NK1.1^–^ cells (4 × 10^5^ to 8 × 10^5^) were injected intrathymically, as described previously (4). Their progeny were analyzed 1 to 4 weeks later, after MACS-enrichment of CD1d-tetramer-positive cells.

## Results

### CD1d Is Required for Thymic Maturation of Positively Selected NKT Cells

**FIGURE 1:**
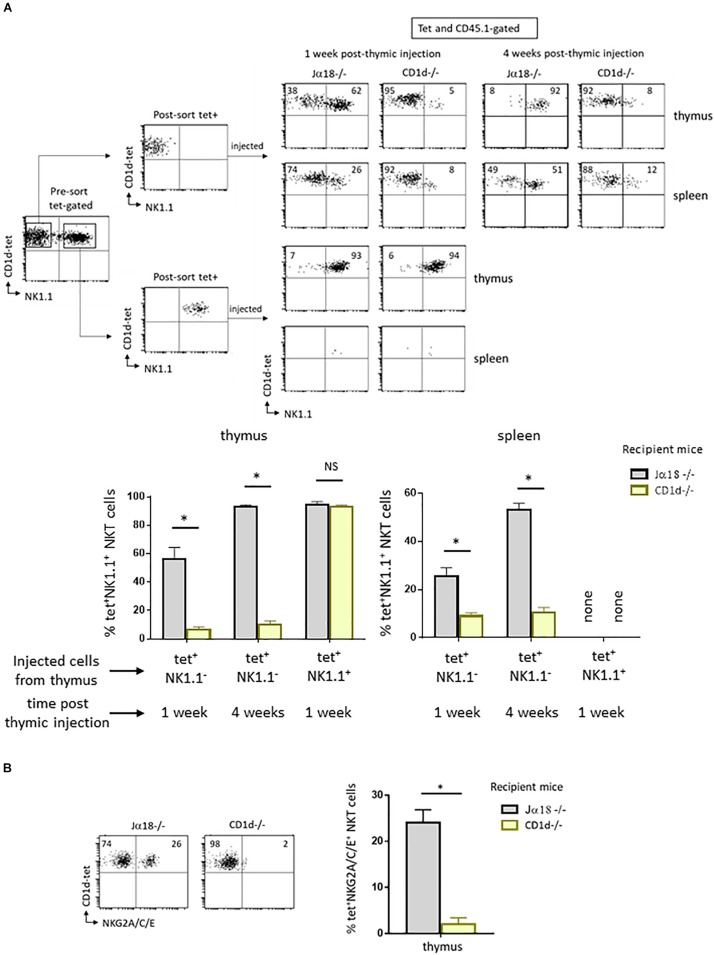
CD1d expression is required for maturation of post-selected NKT cells. **(A)** CD1d-tetramer-positive (tet^+^) and NK1.1 negative (NK1.1^–^), or tet^+^NK1.1^+^ subsets (as gated) were FACS-sorted from CD8-negative thymocyte preparations obtained from 2 to 4 week-old congenic CD45.1 C57BL/6 mice. Cells were injected into the thymus of NKT-deficient CD45.2 Jα18–/– or CD1d–/– recipient mice, both on C57BL/6 background. One or four weeks after injection, expression of the NK1.1 marker was assessed on CD45.1^+^ tet^+^ NKT cells in the thymus and spleen. Histograms show the frequency of tet^+^ NKT cells expressing NK1.1 in various recipient mice, and times. **(B)** Cells were FACS-sorted like in **(A)** and then injected intrathymically into CD45.2 NKT-deficient Jα18–/– or CD1d–/– recipient mice. Three weeks after injection, the expression of NKG2A/C/E was assessed on CD45.1^+^ tet^+^ NKT cells in the thymus of injected mice. Histograms show the frequency of tet^+^ NKT cells positive for NKG2A/C/E. Data are from 3 to 6 experiments, using 4 to 5 recipient mice per experiment. Statistical significance is represented by an asterisk and was evaluated by applying a non-parametric Mann-Whitney *U* test. A *p*-value < 0.05 was considered significant. NS, non-significant. Numbers in dot plots represent percentages of cells in the associated quadrant.

To understand the role of CD1d/Vα14-Jα18/Vβ TCR interaction in NKT maturation following positive selection, we sorted NK1.1^–^ tet^+^ cells from the thymus of wild type C57BL/6 mice expressing the congenic marker CD45.1 and injected them intrathymically into CD1d-deficient (CD1d−/−) mice or CD1d-sufficient (Jα18−/−) mice, both expressing the congenic marker CD45.2. Seven days after injection, we assessed the maturation of tetramer positive (tet+) CD45.1-expressing NKT cells by examining their acquisition of the NK1.1 marker. As shown in the upper panel, Figure 1A, 62% of NKT cells are NK1.1^+^ in Jα18−/− mice, whereas only 5% of NKT cells acquired NK1.1 in CD1d−/− mice, indicating that they remain blocked at the NK1.1^–^ stage. The very low levels of maturation that are still observed in CD1d−/− mice probably result from CD1d/Vα14-Jα18/Vβ TCR interactions between injected CD1d-sufficient NKT cells.

These cells do not acquire other NK markers such as NKG2A, C, or E, expressed before or at the same time as NK1.1. This result suggested that they were unable to enter the NK developmental program, and not simply that they could not specifically express NK1.1 (Figure 1B). When mice were analyzed 4 weeks after thymic injection, the NKT cells analyzed in CD1d−/− mice were also blocked at the NK1.1^–^ stage, with only 8% of NKT cells in these mice expressing NK1.1, in contrast to 92% in Jα18−/− mice (Figure 1A, upper panel). These results rule out the possibility that maturation kinetics are slowed due to the absence of CD1d. Interestingly, we detected tet^+^ cells in the spleen of CD1d−/− injected mice, at both 1 and 4 weeks after thymic injection. Unlike thymic maturation, migration out of the thymus therefore does not appear to be abrogated in the absence of CD1d/TCR interaction (Figure 1A, upper panel).

The absence of CD1d expression may not be the only reason for the block in maturation of NK1.1^–^tet^+^ cells in a CD1d deficient environment. In fact, once injected into CD1d−/− mice, NK1.1^–^tet^+^ cells could reach the NK^+^ stage but then revert to their initial state due to the absence of CD1d expression. To test this possibility, we followed the fate of NK1.1^+^tet^+^ cells isolated from wild type animals after intrathymic injection into CD1d−/− mice. As shown in the lower panel in Figure 1A, NK1.1^+^tet^+^ cells retained their expression of NK1.1 following injection into CD1d−/− mice, and this was also the case when cells were injected into CD1d sufficient Jα18−/− mice (Figure 1A, lower panel). We were unable to detect tet^+^ cells in the spleen of either of these NK1.1^+^tet^+^-injected mice, indicating that mature NK1.1^+^tet^+^ cells do not migrate to the periphery (Figure 1A, lower panel). This result is in agreement with previous findings showing that tet^+^ cells at the NK^–^ stage of development are the ones exiting the thymus to colonize peripheral organs, where they pursue their maturation (4).

**FIGURE 2:**
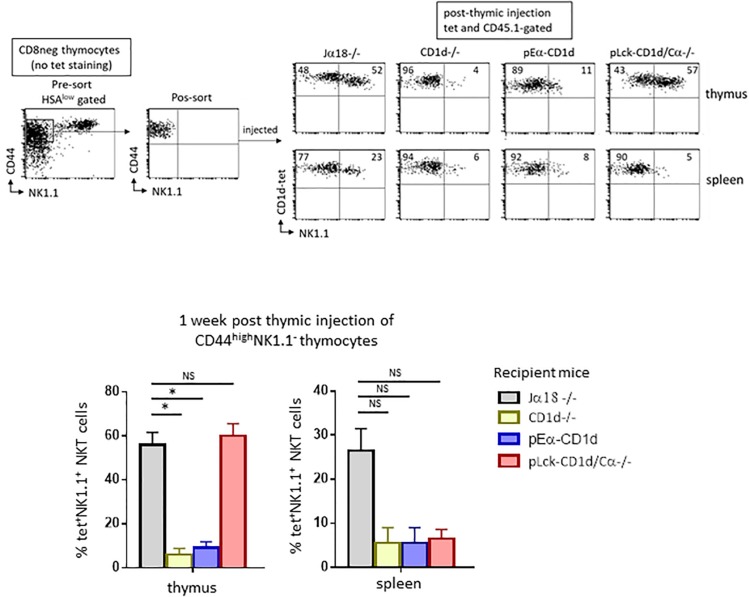
CD1d-expression on cortical thymocytes, but not antigen presenting cells, promotes maturation of NKT cells. CD8-depleted thymocyte preparations from CD45.1 C57BL/6 mice were stained with HSA, CD44, and NK1.1. HSA^low^CD44^high^NK1.1^–^ cells were FACS-sorted for intrathymic injection into CD45.2 NKT-deficient Jα18–/–, CD1d–/–, pEαCD1d or pLck-CD1d/Cα–/– recipients. One week after injection, the expression of the NK1.1 marker was assessed on CD45.1^+^ tet^+^ NKT cells in the thymus and spleen of injected mice. Histograms show the frequency of tet^+^ NKT cells expressing NK1.1 in the different recipient mice. Data are from 4 to 5 experiments, using 3 to 5 recipient mice per experiment. Statistical significance is represented by an asterisk and was assessed by a non-parametric Mann-Whitney *U* test. A *p*-value < 0.05 was considered significant. NS, non-significant. Numbers in dot plots represent percentages of cells in the associated quadrant.

To ensure that TCR engagement by CD1d-αGalCer tetramers during sorting was not responsible for the maturational block observed, we isolated and intrathymically injected sorted thymic CD44^high^NK1.1^–^ cells, the majority of which are NKT cells, into CD1d−/− mice. Seven days after injection, the maturation of CD45.1-expressing NKT cells based on their acquisition of the NK1.1 marker was assessed (Figure 2). The results showed that tet^+^ cells recovered 7 days after thymic injection in CD1d−/− mice barely express NK1.1 when compared to cells injected into Jα18−/− mice (4% vs. 52% of NK1.1^+^ cells, respectively), confirming our hypothesis that the arrest at the NK^–^ stage in CD1d−/− mice is due to the absence of CD1d and not to TCR crosslinking by CD1d-αGalCer tetramers during sorting. In addition, as shown in Figure 2, the CD44^high^NK1.1^–^ intrathymically injected cells can reach the spleen, but those injected into CD1d−/− animals did not progress to the NK^+^ stage as efficiently as those injected into Jα18−/− mice (6% vs. 23% of NKT cells express NK1.1, respectively).

Altogether, these results are in agreement with previous reports (15), and indicate that CD1d/TCR interactions are important for thymic and peripheral maturation of NKT cells. However, these interactions are dispensable for migration of immature NK1.1^–^ NKT cells from the thymus to peripheral organs.

### CD1d-Expression on Cortical Thymocytes but Not Antigen Presenting Cells Promotes NKT Cell Maturation

We next wanted to determine which CD1d-expressing cell type is required for the maturation of NKT cells, so we tested the individual contributions of cortical thymocytes, epithelial cells, dendritic cells, and macrophages in this process. To do so, we used mice with differential CD1d expression. A mouse missing endogenous CD1d (CD1d−/−) where CD1d is transgenically redirected thanks to the MHC class II Eα promoter (pEα-CD1d) was used to test whether epithelial cells, dendritic cells, or macrophages contributed to the maturation of NKT cells (16). Previous studies indicated that this pattern of CD1d expression does not allow NKT cell selection, but whether these cells actually play a determinant role in NKT cell maturation following selection remained to be determined. We therefore injected sorted CD44^high^NK1.1^–^ cells into the thymus of pEα-CD1d mice. The injected cells did not acquire NK1.1 expression as efficiently as in Jα18−/− mice (11% vs. 52% of NKT cells express NK1.1, respectively, as shown in the representative dot plot in Figure 2, upper panel), thus CD1d expressed on presenting cells is not sufficient to promote thymic NKT cell maturation. Tet^+^ cells were detectable in the spleen of pEα - CD1d-injected mice, but like thymic NKT cells, fewer of these cells expressed NK1.1 compared to Jα18−/− mice (8% vs. 23% of NK1.1^+^ NKT cells, respectively, as shown in the representative dot plot in Figure 2, lower panel).

To test whether cortical thymocytes play a role in the maturation of NKT cells, we used mice where CD1d expression is driven by a proximal Lck promoter, where CD1d expression is limited to T cells up to the DP stage of their development (pLck-CD1d mice) (14). Because this CD1d expression pattern supports NKT cell selection, and because we wished to limit CD1d expression to immature T cells, we abrogated T cell development in these mice by crossing them with Cα−/− mice (14). When we injected sorted CD44^high^NK1.1^–^ cells into the thymus of pLck-CD1d/Cα−/− mice, the injected cells converted into NK1.1^+^ cells as efficiently as the cells injected into Jα18−/− mice (57% vs. 52% of NKT cells express NK1.1, respectively, as shown in Figure 2, upper panel). In the spleen, tet^+^ NKT cells were detectable, although they do not progress to the NK^+^ stage, confirming that CD1d is required for peripheral maturation of NKT cells (Figure 2, lower panel).

Together, these results suggest that cortical thymocytes play a crucial role in NKT cell maturation in the thymus and suggest that continuous interaction between NK1.1^–^tet^+^ cells and CD1d-expressing cortical thymocytes is necessary to generate fully mature thymic NKT cells. They also show that peripheral maturation of NKT cells requires CD1d/Vα14-Jα18/Vβ TCR interactions.

### Ligands Required for NKT Cell Selection Are Also Necessary for Their Subsequent Maturation

**FIGURE 3:**
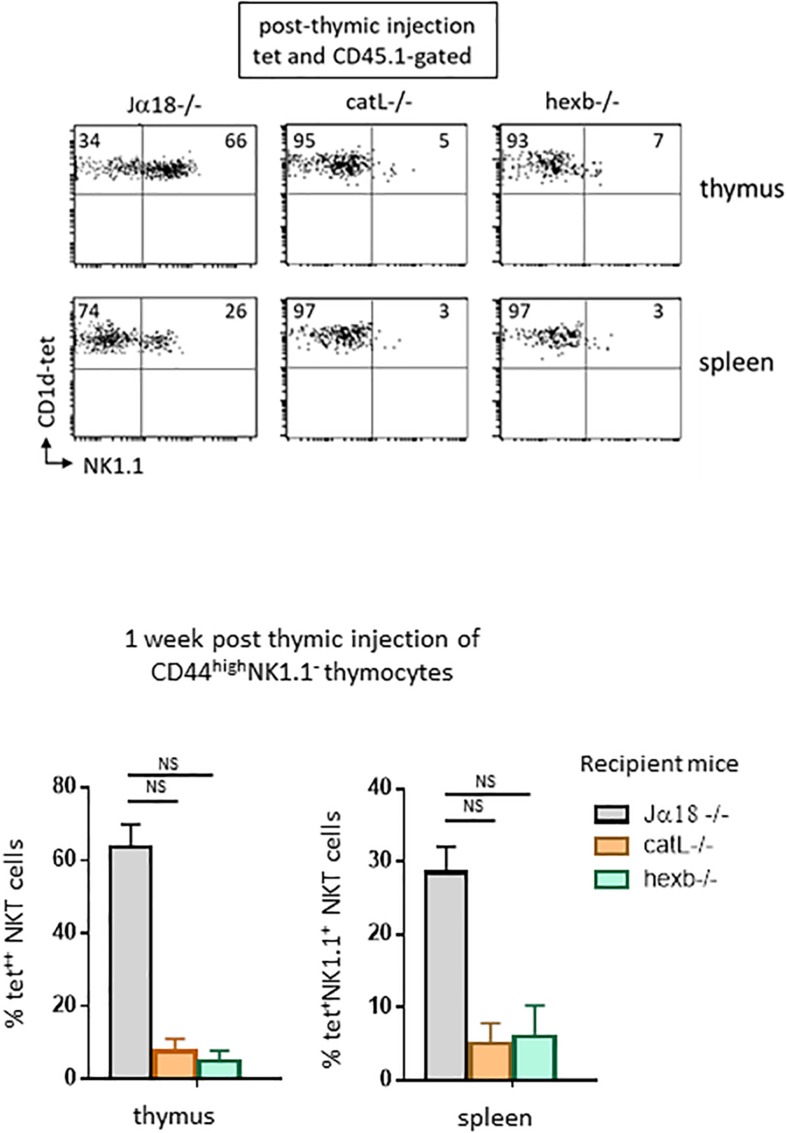
Ligands required for NKT cell selection are also required for subsequent maturation. CD45.1^+^HSA^low^CD44^high^NK1.1^–^ cells were FACS-sorted like in Figure 2, and then injected intrathymically into CD45.2 NKT-deficient Jα18–/–, catL–/– or hexb–/– recipient mice. One week after injection, expression of the NK1.1 marker was assessed on CD45.1^+^ tet^+^ NKT cells in the thymus and in the spleen of recipient mice. Histograms show the frequency of tet^+^ NKT cells expressing NK1.1 in the different recipient mice. Data are from 4 to 5 experiments, using 3 to 5 recipient mice per experiment. Statistical significance was assessed by a non-parametric Mann-Whitney *U* test. A *p*-value < 0.05 was considered significant. NS, non-significant. Numbers in dot plots represent percentages of cells in the associated quadrant.

We next investigated whether ligands required for NKT cell selection are needed for their subsequent maturation. To address this question, we used mice deficient for the arginine endopeptidase cathepsin (catL−/−). In a previous study (17), we showed that the absence of catL expression in thymocytes abrogates their capacity to stimulate autoreactive NKT cells *in vitro* and precludes their selection *in vivo*. CD1d cell-surface expression and intracellular localization were normal in catL-deficient thymocytes, as was lysosomal morphology. These observations suggested a specific role for catL in regulating presentation of natural CD1d ligands mediating NKT cell selection. Sorted CD44^high^NK1.1^–^ cells injected into the thymus of these mice and observed 7 days later revealed that few thymic NKT cells express NK1.1 compared to when cells were transferred into Jα18−/− mice (5% vs. 66% of NKT cells express NK1.1, respectively, as shown in representative dot plots in Figure 3, upper panel). Thus, catL is also important for NKT cell maturation.

The second mice we used lack beta-hexosaminidase b (hexb−/−), an enzyme involved in generating the lysosomal glycosphingolipid isoglobotrihexosylceramide (iGb3) recognized by mouse NKT cells. Impaired generation of lysosomal iGb3 in hexb−/− mice has been shown to result in severe NKT cell deficiency (19). Cortical thymocytes deficient in iGb3 have normal CD1d expression, but do not stimulate autoreactive NKT cells *in vitro*, suggesting that this lipid mediates NKT cell development in mice. To determine whether cortical thymocytes from these mice support the maturation of positively selected NK1.1^–^tet^+^ cells, we injected sorted CD44^high^NK1.1^–^ cells into the thymus of these mice. Seven days later, few cells expressed NK1.1 compared to what was observed in the control Jα18−/− mice (7% vs. 66% of NKT cells express NK1.1, respectively, as shown in representative dot plots in Figure 3, upper panel).

In both catL−/− and hexb−/− mice, NKT cells could migrate to the spleen, but few of these cells acquired NK1.1 expression, indicating similar requirements for peripheral maturation (Figure 3, lower panel). Overall, these results strongly suggest that endosomal ligands required for positive selection also support the maturation of NKT cells, and therefore have an extended role after positive selection.

## Discussion

In this study we used intrathymic injection of sorted “precursor” NK1.1^–^ NKT cells into various genetically modified mouse strains to determine which CD1d-expressing cell type was involved in the maturation of post-selected NKT cells. We found that CD1d expression on cortical thymocytes, but not APC or epithelial cells, is required to drive maturation of already selected NKT1 cells; and that this maturation step is not required for NKT cells to migrate from the thymus to the periphery. We also found that elements involved in regulating the presentation of selecting natural ligands by CD1d have an extended function, as they are also necessary for NKT cell maturation.

**FIGURE 4:**
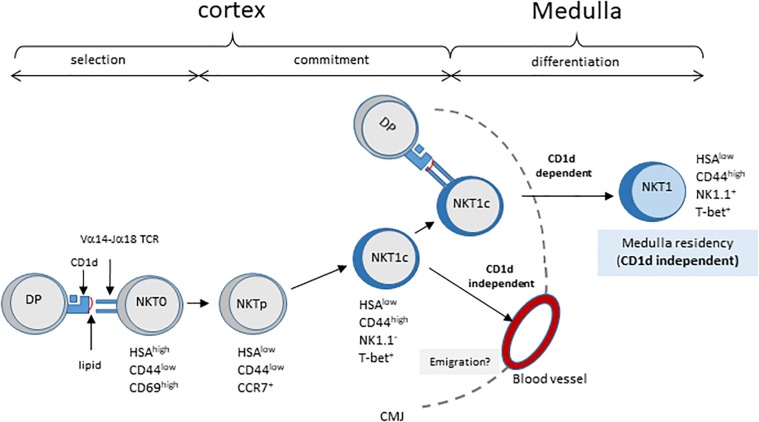
Model of NKT cell development involving CD1d/TCR interaction in post-selection NKT cell maturation. Cortical DP thymocytes expressing the canonical Vα14–Jα18 TCR are selected by CD1d-expressing neighboring DP thymocytes. These HSA^high^ NKT0 NKT cells lose HSA expression and acquire CCR7 expression, giving rise to NKT cell precursors, NKTp. Some NKTp will commit to the NKT1 lineage by expressing T-bet (NKT1c) but no other markers of the NK lineage. During their progression to the cortex, NKT1c cells continuously sense ligands exposed on cortical thymocytes. This sensing is required for their progression to the NK1.1 + NKT1 stage in the medulla, and long-term residency in the thymus. These CD1d/TCR interactions appear not to be essential for NKT1c migration to the periphery. CMJ, cortico-medullary junction.

A study by Lee et al. (20) analyzed the distribution of NKT cell subsets in the thymus, and found that most mature cells reside in the medulla. As the medulla is required for the differentiation of NK1.1^+^ NKT1 cells (21), these authors proposed a model whereby NKT cells migrate to the medulla where they differentiate into various subsets. However, here we injected NK1.1^–^CD44^high^ thymocytes which included T-bet^+^ NKT cells that are committed to the NKT1 differentiation pathway (around 50% of CD44^high^ NKT cells are T-bet^+^, data not shown), and found that these cells require cortical thymocytes for their maturation. These results suggest that immature committed T-bet^+^ NKT1 cells (NKT1c) are located in the cortex, as illustrated in the model depicted in Figure 4. The Vα14-Jα18/Vβ TCR expressed on these proliferating NKT1c cells would undergo successive encounters with CD1d, thus influencing subsequent decisions. For instance, some cells will leave the cortex and progress to the NK^+^ stage to become thymic-resident in the medulla. Based on this model, we would expect to observe maturing T-bet^+^ NKT cells in the cortex by immunofluorescence. In support of this model, Lee et al. (20) effectively observed T-bet^+^ NKT cells in the cortex, and estimated an average of 30% of all NKT cells to be located there, regardless of subset or mouse strain. They proposed that these cells represented a fraction of NKT cells that could have moved back to the cortex after differentiation. A non-exclusive interpretation of these findings could be that these cells include developing committed T-bet^+^ NKT1 cells (NKT1c) passing through the cortex on their way to the medulla. Also, our data show that the migration of NK1.1^–^ cells from the thymus to the periphery occurs even in the absence of CD1d expression on cortical thymocytes. However, our results do not allow us to conclude as to whether this migration should be considered inappropriate – due to insufficient interaction with CD1d in the thymus – or as regular migration but that the lack of CD1d or its ligands in the periphery (expressed on APC, including B cells, or on tissue specific CD1d-expressing cells) prevents further maturation.

Recent studies reported a correlation between NKT cell subsets with differences in the strength of signals received through the Vα14-Jα18/Vβ TCR (6, 22). In fact, high TCR signals are observed in NKT2 cells at the steady state and are thought to originate from continuous TCR signaling, whereas NKT1 cells may have a lower TCR signal threshold, allowing them to survive without continued TCR stimulation. How do these data fit with the findings presented here, as our model proposes that NKT1c experience continuous TCR signaling? One possibility is that TCR signaling strength also depends on the CD1d-expressing cell type interacting with developing NKT cells. As such, cortical thymocytes provide continuous weak TCR signals to developing NKT1c, whereas macrophages provide high TCR signals to NKT2 cells, as proposed by Wang et al. (23). The difference in signaling strength could depend on the nature of ligands presented by CD1d and on additional signals from costimulatory molecules. For instance, these additional signals could be provided to NKT1c cells by SAP-dependent homotypic interactions between SLAM family members; these interactions might still be extensive after positive selection. In support of this hypothesis, recent studies reported that SLAM family receptors (SFR) promote NKT cell development by reducing TCR strength after positive selection, and that lack of SFR alters NKT cell subset distribution, most significantly affecting NKT1 cells (24). Nevertheless, it remains possible that in their early stages of development, potential immature NKT2 precursor/committed cells (NKT2c) are dependent on CD1d expressed on cortical thymocytes for their cortical commitment like NKT1c, and that at later stages they come to rely on medullary APC for steady-state IL-4 production. An accurate characterization of the developmental stages of NKT2 cells could help to test this hypothesis.

The results from our study, though consistent with a failure to generate natural ligands, such as iGb3, do not definitively resolve the mechanisms behind the effect of cathepsin-L and hexosaminidase b knockdown on NKT cell maturation. One possible explanation for the dependence of thymocyte-mediated selection of NKT cells on catL is that catL may regulate the activity of a lysosomal enzyme or enzyme cofactor involved in ligand processing prior to CD1d loading. This regulation could, for example, involve mediation of proteolytic cleavage of the enzymatically inactive prosaposins to generate saposins, suggested to promote loading and editing of lipid ligands on CD1d molecules. In addition to reduced ligand loading on CD1d, the defect in NKT cell development in saposin−/− mice could be due to lipid accumulation, such as that observed in several glycosphygolipid (GSL) storage models, including hexb−/− mice (25). One hypothesis (among others) explaining the NKT cell developmental defect, could be that high levels of storage GSLs outnumber natural ligands, such as iGb3, within the late endosome/lysosome and therefore out-compete the endogenous lipids for CD1d binding. This competition would then affect NKT cell positive selection. Whatever the case, our results underline the importance of integrity of the lysosomal/endosomal compartment for the further maturation of NKT cells.

In summary, this study demonstrated the importance of CD1d expressed on cortical thymocytes in the promotion of NKT cell maturation, and indicated that elements involved in regulating the production and/or presentation of natural selecting ligands have an extended function as they are also necessary for NKT cell maturation.

## Data Availability Statement

The datasets generated for this study are available on request to the corresponding author.

## Ethics Statement

The study was approved by the local Ethics Committee “Comité d’Ethique Paris-Nord; C2EA-121,” affiliated to the “Comité National de Réflexion Ethique en Expérimentation Animale” (CNREEA) and to the French ministry for higher education and research.

## Author Contributions

KB and JK designed, performed, and analyzed the experiments. KB wrote the first draft of manuscript and supervised the study.

## Conflict of Interest

The authors declare that the research was conducted in the absence of any commercial or financial relationships that could be construed as a potential conflict of interest.
